# “It Was Something Outside of Me, Controlling Me”: Young People's Perspectives on the Role of Externalisation in Recovery From Anorexia Nervosa

**DOI:** 10.1002/jclp.70196

**Published:** 2026-07-27

**Authors:** Alex Skolnick, Lucinda J. Gledhill, Rosie Satherley

**Affiliations:** ^1^ Department of Psychology University of Surrey Guildford UK; ^2^ Maudsley Centre for Child and Adolescent Eating Disorders South London and Maudsley NHS Foundation Trust London UK

**Keywords:** adolescent, anorexia nervosa, recovery

## Abstract

**Objective:**

Externalisation is a therapeutic approach that separates the problem from the person, widely used in treating anorexia nervosa (AN) to help young people understand their illness and to collaborate with their families against the disorder. Whilst adult and clinician perspectives on externalisation are well documented, young people's experiences remain underexplored. This study addresses that gap, examining how young people experience AN in relation to their identity and the therapeutic use of externalisation.

**Method:**

Ten older adolescents and young adults (aged: 15–21) who had received treatment for AN or atypical AN as an adolescent, participated in semi‐structured interviews. Interviews were transcribed and recorded verbatim, then analysed using reflexive thematic analysis.

**Results:**

Thematic analysis identified three key themes: “Identity fusion” “Empowerment through externalisation” and “Sustaining the battle.”

**Conclusions:**

Findings suggest that although young people may initially resist externalisation, viewing it as a threat to their identity, the technique helps them and their families better understand the illness, reducing feelings of blame and guilt. Over time, externalisation enables young people to reclaim their identity and redefine themselves beyond AN. The study highlights implications for clinical practice and future research.

## 1 Introduction

Externalisation is a therapeutic technique used across several psychological interventions, most notably in family therapy for anorexia nervosa (FT‐AN; Eisler et al. 2016). First developed within narrative therapy by White and colleagues (White and Epston 1990), externalisation attempts to provide a change in perspective by separating the problem from the person, reducing blame and guilt, and providing a target for treatment (Eisler et al. 2016; Reeves and Sackett 2021).

Externalisation is a core component of FT‐AN, the recommended first line treatment for AN or atypical anorexia nervosa (AAN) in young people (National Institute for Health and Care Excellence 2017; Hilbert et al. 2017). In this model, psychological and behavioural changes are reframed as the effects of AN rather than intrinsic to the individual, enabling parents and clinicians to have a focus for treatment while maintaining a compassionate stance toward the young person. Externalisation is often enacted through metaphorical language to describe the illness as distinct from the person, a concept that resonates with many patients who describe an “anorexic voice,” experiencing intrusive, critical thoughts that feel separate from their true self (Pugh 2020).

However, research suggests that the experience of this “voice” is complex and variable. Phenomenological studies suggest this separation is fluid, as individuals may shift between externalising AN and identifying with it directly (Williams and Reid 2012). Similar processes have been observed in obsessive‐compulsive disorder (OCD), where externalising the illness can help individuals and families separate intrusive thoughts from the self (Banting and Lloyd 2017) but may also create tension if the experience feels internally generated (Coughtrey et al. 2012). These findings highlight that externalisation is not a neutral or universally accepted process, but one that interacts dynamically with identity and self‐concept. Despite its widespread use, young people's experience of externalisation for AN has not been directly explored in research. Broader qualitative research highlights mixed responses to externalisation. For many young people, externalisation can foster a sense of identity, promote recovery, and reduce family conflict (Medway and Rhodes 2016; Conti et al. 2021). However, for some, externalisation can feel disempowering and alienating, leaving them feeling voiceless and disconnected from their own treatment (Conti et al. 2021; Voswinkel et al. 2021). These varied experiences highlight the importance of clinicians understanding the potential benefits and harm of externalisation, particularly as externalisation may impact young people differently depending on their stage of recovery and family involvement.

This study aims to explore young people's experiences of externalisation to gain deeper insight into how it influences identity development and experiences of treatment, with the goal of improving therapeutic approaches for this population.

## 2 Methods

This study was approached from a critical realist perspective, which asserts that, whilst there is an observable reality in the real world, knowledge and understanding of this will necessarily be influenced by social contexts and histories (Maxwell 2012). A reflective journal was kept throughout the research process to document assumptions, emotional responses and emerging interpretations of the data, using reflexivity to make the researchers' subjective stance transparent and recognise themselves as an instrument within the research. This supported critical engagement with the data and enabled the researcher to examine how personal and clinical perspectives may have influenced coding decisions and theme development.

### 2.1 Participants, Recruitment and Data Collection

Eligible participants were between the ages of 12 and 23 years who received a diagnosis of AN or AAN during adolescence and had experienced treatment from a specialist child and adolescent ED service. To reflect the age range of participants, which included both adolescents and young adults, the group is referred to as young people. Participants were required to have been discharged from the service within the past 5 years and to speak English fluently enough to participate in an interview without an interpreter. Individuals were excluded if they had received a diagnosis of a different ED, had only been diagnosed or treated in adulthood, had not received treatment, were still receiving treatment from a specialist child and adolescent ED service, or had been discharged more than 5 years previously. Participants were recruited through an NHS specialist child and adolescent ED service and social media. All young people who met the inclusion criteria in the NHS service and had consented to be contacted about research were invited to participate. The study poster and contact details were also posted on social media. Participants opted in by contacting the researcher to express interest, and those who provided consent/assent completed an online semi‐structured interview. Interviews, conducted by the lead researcher, typically lasted between 40 and 60 min and were recorded to allow verbatim transcription.

A total of ten participants took part, all of whom were white females. The age of participants ranged from 15 to 21 years (mean age = 18.5). A summary of participant demographics is presented in Table 1.

**Table 1:** Participant demographics.

Pseudonym	Age	Gender	Ethnicity	Age of first symptoms	Age of diagnosis	Length of treatment (as reported by participants)	Treatment
Rosie	18	Female	White British	15	16	2 years	FT‐AN^a^ (outpatient)
Lola	15	Female	White British	13	13	3 months	FT‐AN (outpatient)
Ana	18	Female	White European	15	16	1.5 years	FT‐AN (outpatient), RO‐DBT^b^ (individual and group)
Ava	19	Female	White British	13–14	15	2 years	FT‐AN (outpatient), MFT‐AN^c^
Amelia	18	Female	White British	11	12	5 years	FT‐AN (outpatient), Day hospital admission
Georgia	17	Female	White British	15	15	Almost ~2 years	FT‐AN (outpatient)
Maya	17	Female	White British	9–10	13	~3 years	FT‐AN (outpatient), MFT‐AN, Art therapy
Ruby	21	Female	White British	13	17	~2 years	FBT^d^ (outpatient), SSCM^e^
Flora	21	Female	White British	16	17	9 months	FT‐AN (outpatient)
Pearl	21	Female	White British	12	12	8 years	General psych inpatient admission x2, outpatient CBT,^f^ CAT,^g^ family therapy

^a^ FT‐AN refers to Family Therapy for Anorexia Nervosa.

^b^ RO‐DBT refers to Radically Open Dialectical Behaviour Therapy.

^c^ MFT refers to Multi‐Family Therapy for Anorexia Nervosa.

^d^ FBT refers to Family‐Based Treatment.

^e^ SSCM refers to Specialist Supportive Clinical Management.

^f^ CBT refers to Cognitive Behaviour Therapy.

^g^ CAT refers to Cognitive Analytic Therapy.

### 2.2 Interview Schedule

An interview guide developed with guidance from people with current/previous experience of AN, those working in specialist ED services and a previous study looking at adult experiences (Voswinkel et al. 2021) was used for data collection. Interviews began with broad questions about participants' experiences of their ED and their path into treatment, before becoming more focused on their views of how AN relates to their identity and the use of externalisation within treatment. Follow up questions were used to clarify or elicit further exploration. The guide was used flexibly, so that if topics in the guide were raised spontaneously by participants, they could be explored in the moment.

### 2.3 Analysis

Data were analysed using Braun and Clarke's (2019) approach to reflexive thematic analysis (RTA). RTA recognises the active role of the researcher, whose subjective understandings become part of the research process, with codes and themes representing their interpretations and analysis. Analysis followed the 6 phases of RTA as described by Braun and Clarke (2021), though progression through the stages was not linear as the process is recursive and iterative. The lead author became familiar with the data through listening to audio‐recordings and transcribing verbatim. Initial codes were then generated, using both semantic (i.e. descriptive) and latent (i.e. analytic) codes, making note of personal reflections. A predominantly inductive approach was used to analyse data, using open coding to emphasise meaning generated from the data. A deductive approach was adopted only to ensure meanings emphasised were relevant to the research question. By paying attention to commonality, difference, and the relationship between codes within and across transcripts, themes were generated. The author reviewed these themes in relation to the data set and research question, aiming for internal homogeneity and external heterogeneity. Data were constantly revisited, and codes compared across the data to ensure their consistent use. Codes and emergent themes were developed and refined throughout analysis. Themes were then defined and named, and data extracts to illustrate these were identified. The final report was produced, creating a narrative around themes to aid explanation.

## 3 Results

Analysis developed three themes (see Table 2). Each theme captured different aspects of how participants understood the relationship between AN, identity and recovery in relation to externalisation.

**Table 2:** Themes and subthemes.

Theme	Subtheme
1. Identity Fusion:	1.1. Entangled identities 1.2. A spectrum of Identity Positions 1.3 The internal struggle between self and AN
2. Empowerment Through Externalisation:	2.1. Separating AN from the self 2.2. Uniting against AN
3. Sustaining the battle	3.1. Shifting identity over time 3.2. Changing views on externalisation

## 4 Identity Fusion

This theme captured diverse views on the relationship between identity and AN, demonstrating evolving and sometimes contradictory perspectives. Participants varied in the extent to which they experience AN as part of themselves, separate to themselves or fluctuating between these positions. These differing views appeared to influence participants' responses to externalisation techniques.

### 4.1 Entangled Identities

Participants described a range of experiences relating to AN and their identity. Some experienced their identity becoming entangled with AN whose goals they aligned with, losing sense of themselves without the illness: “*it was all of me” (Rosie)*. AN could be a pervasive presence, overshadowing interests, motivation, and personal values: “*I couldn't identify myself without it*” *(Ana)*. Participants described cutting off friends, family, and other interests to pursue restriction and weight loss.

Receiving a diagnosis could reinforce their identification with AN. Rosie reflected that the diagnosis initially felt validating and even rewarding: “*I felt ‘OK, I'm definitely doing it right’*”. Conversely, for others, a diagnosis led to feelings of inadequacy and a sense of disconnection from self: “*it didn't fit with who I felt I was as a person*” *(Flora)*, “*I felt ashamed and embarrassed*” *(Ana)*. Being labelled with AN could be confronting, turning what was once seen as “*my big secret flaw*” *(Flora)* or “*little quirky personality trait*” *(Lola)* into a serious medical issue needing attention. Some participants rejected the diagnosis because they felt so aligned with AN: “*you're being treated for something that you genuinely either don't see as a problem or don't wanna get rid of*” *(Amelia)*. Some did not consider themselves to have a problem and resisted the framing of their desires as an illness.

### 4.2 A Spectrum of Identity Positions

Participants varied in their understandings of AN, ranging from viewing it as an internal manifestation of their thoughts to an external force that had taken over. AN was described through vivid metaphors like a “*demon*” *(Ruby)*, “*parasite*” *(Rosie)* or “*the devil on my shoulder*” *(Maya)*. These metaphors reflected experiences of AN as invasive and overpowering. Participants described feeling “*hijacked*” *(Ruby)* by the illness, leaving them disconnected from their previous selves: “*It was like… a completely different person…I didn't recognise me*” *(Georgia)*.

In contrast, others saw AN as an internal expression of their own thoughts and desires, leading to a fusion of AN and their identity: “*The thoughts sounded like my voice and…is in my head and that's why I was like* ‘*well, if it's in my head, I control it*’” *(Amelia)*. Recognising the thoughts as coming from within their own minds led some participants to view AN as an expression of themselves.

However, many participants occupied a more ambiguous position where AN was both a part of them and distinct from them. Pearl's view encapsulated this complexity: “*I didn't necessarily think* ‘*this is me,*’ *but I also didn't necessarily think* ‘*this is something else like other than me*’.” Participants often moved fluidly between these positions, suggesting that the relationship between identity and AN was dynamic rather than fixed.

### 4.3 The Internal Struggle Between Self and AN

The way participants related to AN and perceived its connection to their identity played a crucial role in shaping their responses to externalisation. Responses ranged from resistance and frustration to cautious recognition of its potential value.

Participants who closely identified with AN often found externalisation invalidating or irrelevant: “*I don't know what you're on about, like these are just my thoughts*” *(Maya)*. They struggled with separating themselves from AN, and so dismissed externalising language: “*In my eyes, I was the same person, and I couldn't differentiate the two*” *(Amelia)*. For some, externalisation created distance between themselves and clinicians or family, increasing feelings of frustration or alienation. Maya reflected on how externalisation “*puts space in between me and the like treatments*”. Participants described feeling that their genuine emotions and beliefs were sometimes dismissed or attributed entirely to AN. Flora compared this experience to “*a weird dystopian simulation*” where her feelings no longer felt recognised as real.

When AN was experienced as intertwined with identity, criticisms of the illness could also feel like criticism of the self: “*I took it personally*” *(Amelia)*. Participants described externalisation as threatening because it implied losing a valued or familiar part of themselves. Rosie compared this process to “*cutting an arm off*” while Ana described feeling “*robbed*”. Several participants expressed fear about who they might become without AN.

Even participants who accepted externalisation sometimes described feeling overwhelmed by the perceived power of AN. Georgia explained: “*when the voice is there, like all the time, it just kind of like wears you down and you just think… it's… easier to just agree with it*.” In these situations, externalising the illness could inadvertently reinforce AN's dominance by highlighting the difficulty of resisting it.

## 5 Empowerment Through Externalisation

The second theme captured how externalisation could be empowering over time. Participants recognised the process of externalisation as instrumental in their recovery, helping them separate AN from their identity and unite with clinicians and family against it.

### 5.1 Separating AN From the Self

Externalisation helped participants view AN as separate from themselves, making the illness and its effects more manageable: “*It was something outside of me, controlling me*” *(Ruby)*. Metaphors and imagery often supported this process. Amelia described using visual representations to distinguish between herself and AN, giving her a clearer sense of what was happening and what the target of treatment was: “*I remember having like something on my wall that was like a devil and then the sunshine*” … “*that was helpful to distinguish the two*”. This also helped participants communicate their experiences more clearly to family members, allowing them to differentiate between their own thoughts and AN's influence. Pearl described referring to AN as “*the bitch in the attic*”, explaining that this gave her family a shared language to understand what she was experiencing.

Seeing AN as an external entity helped participants recognise the control AN was exerting over their thoughts and behaviours. It also helped them feel understood by clinicians and parents, improving their relationships: “*it's like they could see me as me, and then they could see this other thing, like they were actually listening to me and understanding me*” *(Georgia)*. Separating AN from the self also reduced guilt and self‐blame, as “*it was an illness, it wasn't a choice*” *(Maya)*. Flora reflected that externalisation helped her realise “*it's not your fault*”. Participants described feeling relieved that uncharacteristic behaviours could be understood in the context of their illness rather than an expression of their true self. Some participants valued how externalisation helped parents cope with distressing or aggressive behaviours. Pearl described her mother separating hurtful comments from her ‘real’ self, explaining: “*she separated that any time I did anything horrible*” …. “*That wasn't me, that was someone else*”. Attributing blame to AN reduced conflict within the system, supporting family relationships and reducing blame.

### 5.2 Uniting Against AN

Externalisation also facilitated a sense of shared purpose and the hope that recovery was possible. Participants described how externalisation helped families unite rather than be in opposition to one another, providing a sense of hope and ability to act: “*the physical separation of, it's not inside me like I can actively just remove that bit*” *(Pearl)*. By viewing AN as separate from identity, it gave reassurance that “*this isn't gonna last forever*” *(Flora and Maya*). By suggesting that AN was something that could be overcome, participants felt empowered to envision a future without it: “*It gave me, I guess a lot of opportunity to work out who I was without it*” *(Maya)*. Externalising AN supported participants to consider how they could begin to take control back, explore other aspects of their identity and begin to develop a separate identity. They were able to reconnect with interests, relationships and future aspirations that AN had overshadowed.

## 6 Sustaining the Battle

The final theme captured how participants’ relationship with AN and externalisation evolved over time. Recovery was described as a gradual and non‐linear process involving ongoing negotiation between AN‐related thoughts and a developing sense of self beyond the illness.

### 6.1 Shifting Identity Over Time

Participants experienced shifts in their relationship with AN over the course of their illness and recovery, from a dominant aspect of their identity to becoming part of their history. Ana reflected on this evolution: “*At the beginning… it was a huge chunk of my identity*” … “*now I think it is a part of my identity, but not like overwhelmingly*”.

As participants moved forward in their recovery, they reported a reduction in preoccupation with AN thoughts, which seemed to play a central role in lessening the influence of AN on their identities. Learning to recognise AN thoughts helped to distinguish them as separate from their current selves. Maya explained, “*I do now see it is something completely separate from me and also something that I can control*” *(Maya)*.

Despite this, participants were cautious about labelling themselves as “recovered.” Several described recovery as an ongoing process of managing lingering AN thoughts while no longer allowing them to define their identity: “*it's something that I've experienced and it's something that you know every day I have to ignore … But I wouldn't say it's a part of me, as who I am as a person*” *(Rosie)*. These accounts suggest that recovery is viewed as an active and ongoing process, involving developing confidence in managing AN without viewing it as central to identity. Being able to separate AN from their identity appears a crucial evolution to make progress in recovery.

### 6.2 Changing Views on Externalisation

Participants’ responses to externalisation evolved over time. Initially, it might have been resisted due to a strong identification with AN. Rosie recalled responding to her parents by insisting “‘*no, it's me, this is who I am, you're not going to change that*’”. However, as participants progressed in recovery, they began to appreciate the usefulness of separating themselves from AN, though this was not always straightforward. Ana described experiencing both attachment to AN and recognition of the value of externalisation: “*Separating those two things was very helpful. Umm, but at the same time it was kind of like* ‘*no, don't take that away from me*’ … *it was both of these feelings and experiences*”. This ambivalence continued over time, with participants simultaneously wanting to recover whilst being resistant to change.

There was a sense that externalisation could be unhelpful in certain moments, for example during periods of acute distress or conflict, when they felt fully aligned with AN. Flora explained that during: “*an anorexia meltdown*” externalisation became “*defunct*” because separation no longer felt possible. However, participants found externalising could be helpful in the aftermath, allowing retrospective exploration of the incident “*like a debriefing*” *(Flora)* after the emotions had subsided.

Participants described starting to embrace the externalising approach as they progressed in their recovery, as it became both easier and more meaningful: “*the more you want to get better, the easier you find it, to kind of differentiate it from yourself …you can actually see, like that makes sense*” (Georgia). As motivation to change increased, externalisation became more appreciated as a tool, demonstrating the possibility of life without AN.

Externalisation also supported participants in developing identities beyond AN. Amelia described being encouraged in treatment to think about her future aspirations, relationships and values outside the illness. Experiencing moments where AN less dominant provided hope that recovery was achievable. Ruby reflected: “*It felt like that through small steps, I was going to be able to push it out… it wasn't some innate part of myself that would always be there*”.

Overall, participants described their appreciation of externalisation as a “*gradual*” *(Amelia)* and evolving process or “*journey*” *(Maya)* that became more meaningful as recovery progressed. Whilst painful and resisted at times, creating separation between the self and AN appeared to support hope, agency and the development of a broader identity beyond the illness.

## 7 Discussion

Previous research has examined externalisation in adults with AN (Voswinkel et al. 2021; Cripps et al. 2024), but this study provides novel insights into how externalisation affects young people's sense of self and agency during treatment. The findings align with existing literature on the role of identity in the development, maintenance, and recovery from AN (Croce et al. 2024; Voswinkel et al.^2021^), whilst making a unique contribution by focusing on young people's experiences. Recognising the influence of AN and fostering an internal locus of control have been identified as critical turning points in recovery (Dawson et al. 2014), echoed by the current study which suggests externalisation fosters empathy, reduces blame, and builds a strong alliance between young people and their families.

The theme **Identity Fusion** illustrated the extent to which AN can be experienced as psychologically intertwined with the self, making it difficult to distinguish personal values, emotions, or desires from those associated with the illness. This aligns with literature suggesting that AN can dominate one's self‐concept (Cripps et al. 2024; Pugh 2020; Tan et al. 2003; Williams and Reid 2012) and theories of illness and identity, where illness can be experienced as an “alien force” that threatens the sense of self (Charmaz 1995, p.662). At the same time, the relationship between AN and identity was rarely fixed or absolute, moving fluidly between an external controlling force and an internally generated part of themselves. This complexity echoes phenomenological work on the “anorexic voice” (Pugh 2020) and aligns with findings from OCD research where intrusive experiences may be simultaneously recognised as ego‐dystonic yet experienced as internal and personally meaningful (Coughtrey et al. 2012). Families can similarly find it hard to distinguish the disorder from the identity (Pedley et al. 2017). Like individuals with OCD, participants in the current study appeared to negotiate ongoing tensions between experiences of ownership, control, and alienation in relation to illness‐related thoughts.

Young people's shifting perception of AN, from a positive to a destructive force, yet one they are sometimes reluctant to let go of, underscores the fluid relationship they have with the illness. This change may represent a narrative transformation, where AN shifts from being central to their identity to playing a less dominant role, helping them reclaim their authentic selves, appearing similar to adults’ experiences (Cripps et al. 2024).

The theme **Empowerment through externalisation** further illustrated how separating AN from the self helped young people regain agency, reduce self‐blame, and define themselves beyond the illness. This resonates with findings from Medway and Rhodes (2016) and Rienecke and Le Grange (2022) who found that externalisation can foster empathy and reduce blame within family dynamics. The ability to view AN as an external entity seems to provide a narrative shift that helped young people reclaim agency and redefine their identity beyond AN (Conti 2018).

Externalisation is a flexible technique that can be adapted across therapeutic modalities for example, FT‐AN (Eisler et al. 2016), Maudsley Anorexia Nervosa Treatment for Adults (MANTRA; Schmidt et al. 2014), and schema therapy (Pugh and Rae 2019). In the current study, all participants had experienced externalisation as part of their treatment, and their responses appeared to evolve over time. The current findings highlight important tensions with externalisation practices, as described in the theme **Sustaining the Battle.** Some participants felt that it could inadvertently reinforce the illness's dominance or provide AN with more power by suggesting an external locus of control that might affect motivation to change (Dawson et al. 2014). The findings therefore suggest that externalisation should not be conceptualised as a universally neutral or beneficial intervention, but rather as a therapeutic process that interacts with developmental stage, identity formation and readiness for change.

Importantly, participants’ experiences of externalisation appeared to evolve over time. The findings reinforce the importance of understanding externalisation as one component within a broader therapeutic process. Externalisation appears to become more meaningful alongside other changes, including nutritional rehabilitation, increased motivation for change, supportive family relationships and opportunities to reconnect with or discover valued aspects of identity.

## 8 Clinical Considerations

The findings have several important implications for clinical practice. First, they suggest that clinicians should adopt a flexible and collaborative approach to externalisation. Participants varied considerably in how they understood AN, and externalisation could be validating for some whilst experienced as alienating or dismissive by others. This finding supports concerns raised by Voswinkel et al. (2021) regarding testimonial injustice, where externally imposed illness narratives may undermine individuals lived experience. To mitigate this, clinicians should avoid forcing metaphors, such as the “AN voice,” and instead use language that resonates with the young person's own perspective (Graham et al. 2019). Engaging the young person in co‐creating these metaphors may reduce resistance and foster a greater sense of agency (Cripps et al. 2024; White 2011). Applying externalisation tentatively, considering the young person's stage of treatment and their evolving relationship with AN, can demonstrate respect for the young person's voice.

Clinicians should also remain mindful of the language used within externalisation practices. While combative metaphors such as “fighting” or “defeating” AN may feel empowering for some, they can unintentionally reinforce the dominance of AN, evoking feelings of inadequacy or defeat. Similar concerns have been raised both within ED and narrative therapy literature (White 2007; Wufong et al. 2019). Clinicians should tailor externalisation to the individual and their stage of treatment, ensuring that empowering language is used to avoid eliciting feelings of hopelessness or disempowerment (Cripps et al. 2024; White 2007; Wufong et al. 2019). Therapeutic language that emphasises gradual differentiation may therefore be more helpful than approaches that inadvertently position the young person in an unwinnable ‘battle’ against themselves.

## 9 Strengths, Limitations and Future Directions

This study's strength lies in its focus on discharged patients, providing a retrospective view of how externalisation perceptions changed over time. However, this retrospective approach may introduce recall bias (Westwood and Kendal 2012), and future research should consider longitudinal designs to capture changes during different treatment phases.

There are several limitations to this study, which offer directions for future research. The sample was largely homogenous (white, cisgender females), a common limitation in eating disorder research (Dimitropoulos et al. 2024). AN differentially affects gender‐diverse people and males, who often develop and experience the illness differently (Arnow et al. 2017; Mittertreiner et al. 2024; Murray et al. 2016). Future research should seek more diverse samples to understand how factors like gender, race, and cultural background influence responses to externalisation. Additionally, it would be valuable to explore the effectiveness of externalisation techniques among bilingual or non‐English‐speaking populations, as language and cultural differences may affect the applicability of these metaphors.

Further research into experiential externalisation techniques, such as chair work, may also yield important insights. Chair work allows patients to dialogue with different parts of the self, fostering metacognitive insight and self‐compassion (Pugh 2020; Bell et al. 2020). This technique may be particularly useful for individuals with AN by helping them differentiate and integrate aspects of their self‐concept, thereby supporting recovery (Chua et al. 2022; Hibbs et al. 2021). Future research should explore the applicability of chair work and other experiential externalising techniques in treating young people with AN.

This study provides new insights into how externalisation interacts with young people's sense of self in AN treatment. While externalisation can be a powerful tool in fostering recovery, it must be applied carefully to avoid reinforcing AN's dominance or creating resistance. Future research should address the limitations and challenges of the technique noted. Understanding these dynamics can help clinicians refine externalisation strategies to better support young people's recovery from AN.

## Funding

The authors have nothing to report.

## Ethics Statement

Ethical approval was obtained from Camden & Kings Cross NHS Research Ethics Committee (22/LO/0861).

## Consent

The participants 16 and above provided informed consent, those below 16 provided assent, with consent given on their behalf from a parent.

## Conflicts of Interest

The authors declare no conflicts of interest.

## Data Availability

The data that support the findings of this study are available from the corresponding author upon reasonable request.
